# The association between psychotic-like experiences and violent behavior in adolescents: a cross-lagged longitudinal study

**DOI:** 10.1007/s00787-025-02770-1

**Published:** 2025-06-11

**Authors:** Rui Zhou, Jerome Clifford Foo, Asuka Nishida, Sayoko Ogawa, Fumiharu Togo, Tsukasa Sasaki

**Affiliations:** 1 Department of Physical and Health Education, Graduate School of Education, The University of Tokyo, 7-3-1 Hongo, Bunkyo-ku, Tokyo, 113- 0033 Japan; 2 Department of Psychiatry, College of Health Sciences, University of Alberta, Edmonton, Canada; 3 Neuroscience and Mental Health Institute, University of Alberta, Edmonton, Canada; 4 Department of Genetic Epidemiology in Psychiatry, Medical Faculty Mannheim, Central Institute of Mental Health, University of Heidelberg, Mannheim, Germany; 5 Institute for Psychopharmacology, Medical Faculty Mannheim, Central Institute of Mental Health, University of Heidelberg, Mannheim, Germany; 6 Unit for Mental Health Promotion, Research Center for Social Science and Medicine, Tokyo Metropolitan Institute of Medical Science, 2-1-6 Kamikitazawa, Setagaya-ku, 156-8506 Tokyo, Japan

**Keywords:** Psychotic-like experiences, Violent behavior, Adolescents, Random intercept cross-lagged panel model, Longitudinal study

## Abstract

Psychotic-like experiences (PLEs) have been identified as risk factors for mental health issues and behavioral problems including violence. While cross-sectional studies suggest an association between PLEs and violent behavior in adolescents, their longitudinal relationship remains unexamined. This study aims to examine the temporal association between PLEs and violent behavior in adolescents. PLEs and violent behavior were assessed using data from self-report surveys conducted from 2011 to 2019 in a Tokyo junior and senior high school (grades 7–12). The study included 1685 participants aged 12–18 surveyed annually for up to 6 years. Random intercept cross-lagged panel models (RI-CLPMs) were used to examine between-person and within-person associations among study variables, with analyses stratified by gender. Results showed a bidirectional relationship between PLEs and violent behavior on both the between-person (*β* = 0.23, *p* < 0.001) and within-person levels (*β* = 0.07–0.25, *p* < 0.05). This relationship was significant for PLEs and violence towards objects (between-person: *β* = 0.22, *p* < 0.001; within-person: *β* = 0.07–0.32, *p* < 0.05), but not for PLEs and interpersonal violence. When analyzed by gender, these associations were significant in girls but not in boys. The findings suggested that PLEs may have a bidirectional relationship with violent behavior (especially violence towards objects), particularly in girls, indicating potential gender-specific pathways in this association. Further research should explore the underlying mechanisms of this bidirectional relationship, with a focus on gender-specific factors.

## Background

Psychotic-like experiences (PLEs), encompassing hallucinatory perceptions and delusional thoughts, are believed to exist on a continuum, ranging from nonclinical occurrences in the general population to severe presentations indicative of psychotic disorders [1]. A meta-analysis estimated the median lifetime prevalence of PLEs to be 7.2% in the general population [2]. PLEs are more common in adolescents and children, with the median prevalence rates ranging between 7.5% and 17% [3, 4].

Although previous research has identified a modest increase in the risk of violent behavior among individuals with psychotic disorders [5, 6], they are more likely to be victims rather than perpetrators of violence [7, 8]. However, most of this evidence is drawn from clinical adult populations, leaving it unclear whether similar associations extend to subclinical symptoms—such as PLEs—especially among adolescents. This question is particularly important given that PLEs are relatively common during adolescence and may reflect a period of heightened vulnerability to both internalizing and externalizing problems [3, 9]. Few studies have directly examined the contribution of PLEs to violent behavior in non-clinical samples [10–12].

Youth violence, defined as interpersonal aggressive behavior toward others, oneself, or property [13], is a significant public health issue. It is the fourth leading cause of death in young people worldwide, resulting in approximately 200,000 deaths annually [14]. Violence typically peaks during late adolescence and early adulthood and manifests in various forms, including homicide, assault, bullying, fighting, and violence towards objects (e.g., damaging property or throwing objects). Beyond fatalities and injuries, youth violence has long-term consequences, contributing to chronic physical and mental health issues [14]. Despite its severe impact, relatively few studies have specifically focused on identifying the risk factors that contribute to violent behavior.

Given the high prevalence of PLEs in adolescents and emerging evidence suggesting a possible link between PLEs and violence [3, 10–12], further investigation into this association is crucial. However, most existing studies are cross-sectional designs, which limited our understanding of the temporal sequence of this relationship [10, 11, 15, 16]. Although a longitudinal study in adults indicated an increased risk of violence in individuals with PLEs [12], similar research among adolescents remains scarce. Understanding this association could inform early intervention strategies, such as developing screening tools to identify at-risk adolescents and designing mental health programs to mitigate PLEs and reduce violence. Additionally, while gender differences in violent behavior have been well-documented, with males generally showing higher prevalence [11, 17, 18], findings on gender differences in PLEs are mixed [19–23]. Whether the PLEs-violence relationship differs by gender is unclear and requires further examination.

The present study aims to investigate the longitudinal relationship between PLEs and violent behavior in adolescents. Specifically, we explore whether PLEs precede subsequent violent behavior and whether violent behavior can serve as an indicator of underlying PLEs. We also seek to examine potential sex differences in this relationship, recognizing not only the differing prevalence rates of PLEs and violent behavior between boys and girls [16, 24], but also the possibility that the mechanisms linking PLEs to violent behavior—such as emotional regulation, cognitive function, and responses to distress—may vary by sex and influence how violence manifests in the context of PLEs [25–27]. The random intercept cross-lagged panel model (RI-CLPM) was employed to provide a more nuanced understanding of the longitudinal relationship between PLEs and violent behavior, by differentiating between-person associations from within-person effects.

## Methods

### Participants

The data collection method has been described in our previous work [28]. In brief, data were obtained from a longitudinal survey of adolescent mental health status conducted from 2011 to 2019 in a combined junior and senior high school (grades 7–12) in Tokyo, Japan. The survey was conducted annually in April, and the number of participants ranged from 699 to 707 (participation rate: 97.7–99.0%) each year, with a total of 1685 students (ages 12–18) participating through the course of the study (836 boys and 844 girls, 5 not indicated). After excluding the 5 individuals without sex information, data of 1680 participants with up to six timepoints of data were included in the final analysis. Sex was assessed via a self-report questionnaire item asking participants, “What is your sex?” with the possible response options being “1. Male” and “2. Female”. No options regarding gender identity were provided.

The study was approved by the Ethics Committee of the Life Science Committee of the University of Tokyo (*#15–128*) and by the Research Department of the school. Consent for participation was obtained through a two-step process. First, parental consent was requested via an opt-out system, where parents received a consent form and could return it if they did not wish for their child to participate. Following parental consent, students were asked to provide their own assent. Students who chose not to participate were not required to complete the study.

### Measures

PLEs occurring in the past 6 months were assessed using five items from the Schizophrenia section of the Japanese version of the Diagnostic Interview Schedule for Children (DISC-C) [29]. Two items assessed hallucinatory experiences (auditory and visual) and three items assessed delusional experiences: (1) thoughts being read, (2) feeling spied upon, and (3) receiving special messages. All answers were given on a four-point scale: ‘no’, ‘maybe’, ‘yes, once’, and ‘yes, twice or more’. We defined students who answered ‘yes, once’ or ‘yes, twice or more’ on any of the five items as having experienced PLEs in the survey year.

Violent behavior was assessed using the same questionnaire, which included questions about interpersonal violence (IV) and violence toward objects (VTO) over the past year. The specific items were: ‘Have you physically abused someone in your family or among your friends?’ (IV) and ‘Have you been extremely frustrated and damaged something?’ (VTO). Responses were recorded in a binary format: ‘yes’ or ‘no’. This measure has been used in a previous study [11].

### Statistical analysis

Descriptive statistics, including the prevalence of PLEs and violent behaviors (IV and VTO) across grades, were calculated. To investigate the associations among variables PLEs, IV, and VTO, as well as their correlations with gender, we employed Generalized Estimating Equations (GEE). GEE is a statistical approach designed to account for within-subject correlations in longitudinal data, ensuring robust parameter estimates.

**Fig. 1:**
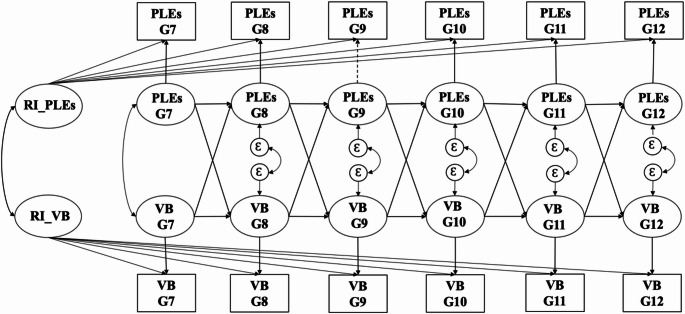
Conceptual random intercept cross-lagged panel model examining the relationship between PLEs and violent behavior. *Note*: PLEs, psychotic-like experiences; VB, violent behavior; G7 − G12, grade 7 to grade 12; RI, random Intercepts; Ɛ: residual variable. All factor loadings are constrained to 1. The squares represent observed variables, and the ovals represent latent variables. Correlations are represented by bidirectional arrows, and regression parameters are represented by unidirectional arrows

The longitudinal relationship between PLEs and violent behaviors was analyzed using random intercept cross-lagged panel models (RI-CLPM, see Fig. 1). Unlike traditional cross-lagged models, RI-CLPMs introduce random intercepts to account for stable between-person differences and allow for the separation of within-person effects from between-person effects [30]. Specifically, RI-CLPMs decompose each observed variable into two components: a between-person component and a within-person component. The between-person component reflects stable, trait-like individual differences in average levels of PLEs and violent behavior over time, represented by two latent random intercepts. The within-person component captures time-specific deviations from these individual averages and includes two types of paths: autoregressive paths and cross-lagged paths. The autoregressive paths examine whether a deviation in an individual’s PLEs (or violent behavior) at one timepoint predicts a similar deviation in the same variable at the next timepoint. The cross-lagged paths, on the other hand, assess whether deviations in PLEs at one timepoint predict subsequent deviations in violent behavior at the next timepoint (and vice versa), controlling for prior levels. This modeling approach enables the examination of temporal dynamics between PLEs and violent behavior at the individual level, independent of stable trait-like differences.

The analysis was conducted using the weighted least square mean and variance (WLSMV) estimator in Mplus 8.11 [30], which is suitable for modeling categorical or ordered data without assuming normal distribution [31]. To identify the most parsimonious model, we tested two versions of the RI-CLPM. Model 1 was a fully constrained (time-invariant) model, in which all autoregressive and cross-lagged paths were constrained to be equal across time points—assuming that the strength of these relationships remains constant year over year. In contrast, Model 2 was a fully unconstrained (time-varying) model, allowing these paths to vary freely over time—thus capturing possible fluctuations in the relationships (e.g., over different developmental stages). Comparing these models allowed us to evaluate whether a simpler model with stable effects over time sufficiently captured the data, or whether allowing temporal variation improved model fit. Model fit was assessed using chi-square (χ^2^), comparative fit index (CFI), root-mean-square error of approximation (RMSEA), and standardized root mean squared residual (SRMR). A model is considered acceptable if CFI > 0.90, and RMSEA and SRMR < 0.08 [32]. When the fit of the constrained model was not significantly worse than that of the unconstrained model, the constrained (i.e., more parsimonious) model was retained. Model comparisons were based on changes in CFI. When △CFI < 0.010 and △RMSEA < 0.015 between the constrained and unconstrained models, the null hypothesis of invariance was accepted and the constrained model was chosen [33]. Cross-lagged effects were categorized as small (0.03 to 0.07), medium (0.07 to 0.12), and large (greater than 0.12) [34]. Statistical significance was determined using two-tailed *p*-values < 0.05.

We first examined the relationship between PLEs and violent behavior, where violent behavior was treated as a combined variable encompassing both IV and VTO. Next, we separately analyzed the relationship between PLEs and each specific type of violence (IV and VTO). Finally, these analyses were conducted separately for boys and girls.

## Results

### Descriptive statistics

Table 1 shows the prevalence of PLEs and violent behavior in adolescents. PLEs were significantly associated with both VTO (coefficient = 1.01, *p* < 0.001) and IV (coefficient = 0.62, *p* < 0.001). Additionally, VTO showed a significant association with IV (coefficient = 1.43, *p* < 0.001). Regarding the prevalence rates, both PLEs and IV were significantly higher in girls than in boys (*p* < 0.001), whereas no significant gender difference was observed for VTO.

**Table 1:** The past year prevalence (%) of PLEs and violent behavior in adolescents in grade 7 to 12

	Whole sample (*N* = 1680)			Boys (*N* = 836)			Girls (*N* = 844)		
	PLEs	VTO	IV	PLEs	VTO	IV	PLEs	VTO	IV
Grade 7	10.8	29.5	30.2	9.1	26.8	34.1	12.5	32.1	26.3
Grade 8	10.3	30.2	27.6	9.1	28.6	32.6	11.5	31.8	22.5
Grade 9	6.5	26.7	18.9	4.4	28.3	21.8	8.7	25.1	16.1
Grade 10	6.5	23.7	14.4	5.3	26.4	17.6	7.7	21.1	11.4
Grade 11	7.0	23.4	11.8	5.2	26.2	14.4	8.7	20.6	10.3
Grade 12	6.6	22.2	11.4	5.2	23.0	11.1	8.1	21.3	11.6

*Note:* PLEs, psychotic-like experiences; VTO, violence towards objects; IV, interpersonal violence

### RI-CLPMs between PLEs and violent behavior

As shown in Table 2, model fit of the fully constrained and fully unconstrained models was acceptable, with no significant differences between them (i.e., △CFI < 0.010, △RMSEA < 0.015). Based on the principle of model parsimony, the fully constrained models were selected as the final RI-CLPMs.

**Table 2:** Fit statistics for the RI-CLPMs between PLEs and violent behavior

Variables	Models	χ^2^ (df)	CFI	RMSEA	SRMR	$\triangle \mathrm{CFI}$	$\triangle \mathrm{RMSEA}$
PLEs and VB	Model 1	75.411 (48)	0.989	0.018	0.065	-	-
	Model 2	36.643 (27)	0.996	0.015	0.046	0.007	-0.003
PLEs and VTO	Model 1	65.500 (48)	0.993	0.015	0.057	-	-
	Model 2	25.459 (27)	1.000	0.000	0.034	0.007	-0.015
PLEs and IV	Model 1	60.568 (48)	0.994	0.065	0.012	-	-
	Model 2	23.134 (27)	1.000	0.000	0.042	0.006	-0.065

*Note:* Model 1 = constrained model, Model 2 = unconstrained model. RI-CLPM, random intercept cross-lagged panel model; PLEs, psychotic-like experiences; VB, violent behavior (violence towards objects and/or interpersonal violence); VTO, violence towards objects; IV, interpersonal violence; df, degree of freedom; CFI, comparative fit index; RMSEA, root mean square error of approximation; SRMR, standardized root mean square residual

**Fig. 2:**
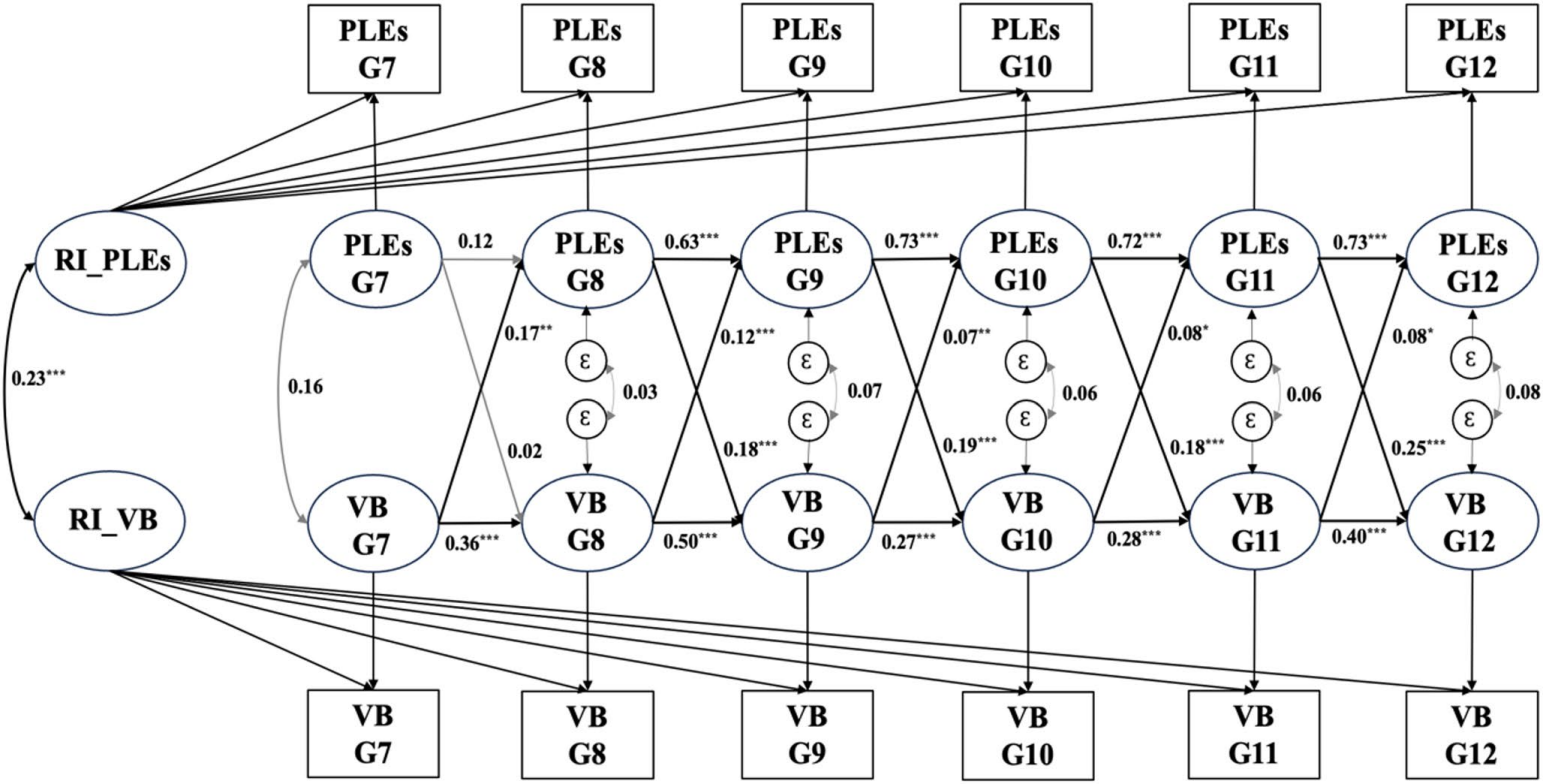
Standardized path coefficients in RI-CLPM for PLEs and violent behavior in whole sample. *Note*: PLEs, psychotic-like experiences; VB, violent behavior (violence towards objects and/or interpersonal violence); G7 − G12, grade 7 to 12; Ɛ, residual variable; Arrows in bold indicate significant paths. ^∗∗∗^*p* < 0.001. ${ }^*{}^* p<0.01$. ${ }^* p<0.05$

Figure 2 presents the standardized path coefficients of the RI-CLPM between PLEs and violent behavior. At the between-person level, the correlation between the random intercepts of PLEs and violent behavior was statistically significant, positive, and moderate in effect size (*β* = 0.23, *p* < 0.001); that is adolescents with PLEs were more likely to engage in violent behavior and vice versa. At the within-person level, PLEs displayed statistically significant bidirectional associations with violent behavior across timepoints (*β* = 0.07–0.25, *p* < 0.05). In addition, the autoregressive effects of PLEs and violent behavior were both positive and statistically significant (*p* < 0.001), except for the autoregressive effect of PLEs from grade 7 to grade 8, which was not significant.

**Fig. 3:**
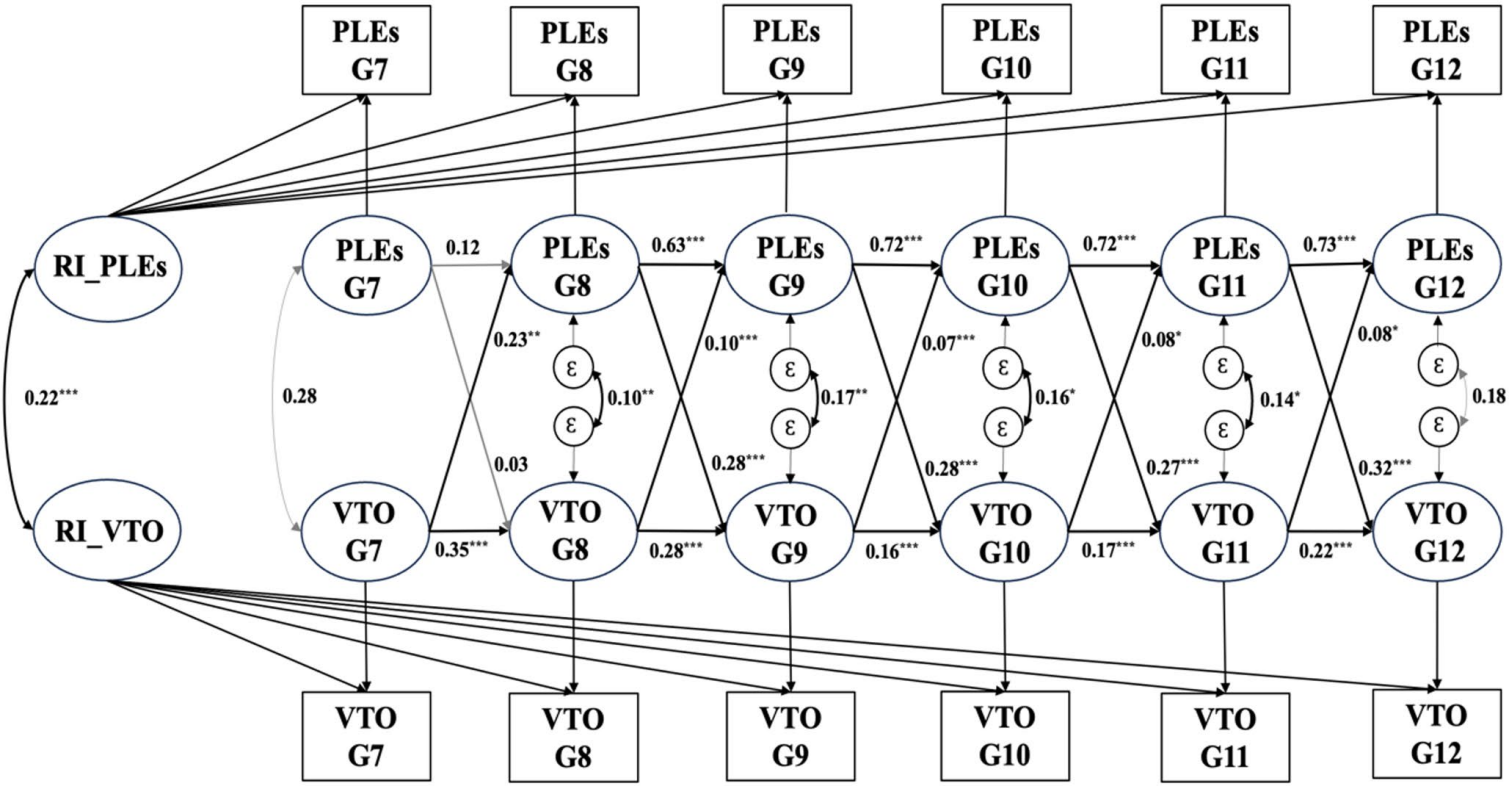
Standardized path coefficients in RI-CLPM for PLEs and violence towards objects in whole sample. *Note*: PLEs, psychotic-like experiences; VTO, violence towards objects; G7 − G12, grade 7 to12; Ɛ, residual variable; Arrows in bold indicate significant paths. ${ }^*{}^*{ }^* p<0.001$, ${ }^*{}^* p<0.01$, ${ }^* p<0.05$

When examining the relationships between PLEs with specific types of violent behavior, different patterns emerged. At between-person level, as shown in Fig. 3, PLEs were significantly correlated with VTO (*β* = 0.22, *p* < 0.001). At the within-person level, the cross-lagged coefficients between PLEs and VTO were statistically significant and positive in both directions (*β* = 0.07–0.32, *p* < 0.05). However, the RI-CLPM estimates for the relationship between PLEs and IV were not statistically significant at either the between-person or within-person levels.

The gender stratified analysis showed that the relationship between PLEs and violent behavior differed in boys and girls. Among girls, the bidirectional association between PLEs and violent behavior were statistically significant at both the between-person level (*β* = 0.23, *p* < 0.05) and the within-person level (*β* = 0.09–0.31, *p* < 0.05). Specifically, there was a significant bidirectional relationship between PLEs and VTO in girls (between-person level: *β* = 0.24, *p* < 0.01; within-person level: *β* = 0.10–0.39, *p* < 0.05), while the association between PLEs and interpersonal violence was not statistically significant at either level. In contrast, among boys, the results of the RI-CLPMs showed no statistically significant associations between PLEs and either type of violent behavior.

## Discussion

The present study is the first to examine the directionality of the association between PLEs and violent behavior in adolescents using a RI-CLPM with multi-timepoint individual data. The results indicate a mutual positive prediction between PLEs and violent behavior at both the between-person and within-person levels. This bidirectional association was particularly pronounced between PLEs and violence towards objects, rather than interpersonal violence. Closer examination with the stratified analysis found that the association was statistically significant in girls, but not in boys, warranting further investigation into gender-specific factors.

These findings align with previous cross-sectional studies linking PLEs to violent behavior [10, 11, 35]. Using RI-CLPM, this longitudinal study provides new insights into the reciprocal nature of the relationship between PLEs and violent behavior by disentangling stable between-person effects from dynamic within-person changes. Our results suggest that adolescents with PLEs are consistently more likely to engage in violent behavior compared to those without PLEs, indicating a pattern that suggests a stable between-person association. Moreover, within-person fluctuations in PLEs correspond with changes in violent behavior, demonstrating a dynamic bidirectional relationship where increases in one are associated with increases in the other. These findings extend previous research by showing that even mild forms of psychotic symptoms (i.e., PLEs) in adolescents may lead to violent behavior, consistent with findings in both clinical and non-clinical adult samples [12, 16, 36]. Early detection and targeted interventions, such as school-based screening and mental health support, could help reduce violent behavior among adolescents by addressing the distress associated with PLEs [37].

There are a number of possibilities which may characterize this bidirectional relationship. Firstly, increases in PLEs might heighten vulnerability to violent behavior through heightened threat perception or impaired self-regulation. Cognitive biases associated with PLEs, such as hostile attribution bias and sensitivity to perceived threats, have been linked to aggression [38, 39]. Stress-induced impairments in self-regulation may further exacerbate violent tendencies in individuals experiencing PLEs [40, 41]. Conversely, violent behavior might intensify PLEs by increasing chronic stress, social rejection, or neurobiological dysregulation. Elevated stress levels, which are associated with hypothalamic-pituitary-adrenal (HPA) axis dysfunction, may heighten susceptibility to PLE [42, 43] Future research should explore the potential mechanisms underlying the relationship between PLEs and violent behavior.

Our study contributes to the existing literature by showing that PLEs are significantly associated with subsequent VTO but not with IV, and suggests that the mechanisms linking PLEs to violent behavior may differ based on the target. Cognitive distortions, such as paranoid thoughts, and emotional dysregulation, like heightened emotional reactivity, are known risk factors for externalizing behaviors [44, 45]. These internal states may lead adolescents to externalize their distress by damaging objects, providing an immediate and solitary outlet for frustration [46]. In contrast, IV often involves specific social or situational triggers, such as peer conflicts or familial dysfunction, where external pressures may play a more dominant role than internal states alone [47, 48]. While PLEs may link to emotional dysregulation or cognitive distortions, these factors are usually insufficient to provoke IV without external provocations [49–51] This distinction highlights that violent behaviors influenced by PLEs vary in their underlying mechanisms, underscoring the importance of first clarifying these differences and then developing targeted interventions to address them.

The gender-stratified analysis indicated that the significant bidirectional association between PLEs and violent behavior was driven primarily by girls, with no significant relationship observed in boys. Specifically, the relationship between PLEs and VTO was statistically significant in girls, whereas the PLEs-IV relationship was not. These differences may be influenced by gender-specific coping mechanisms. Research suggests that girls are more likely to externalize distress through emotional expression or aggression when experiencing cognitive distortions or emotional dysregulation linked to PLEs, particularly in solitary contexts such as VTO, which does not depend on social triggers [52, 53]. In contrast, boys might channel PLEs through risk-taking or other behaviors not directly assessed in this study, potentially explaining the lack of significance in boys [54, 55]. These findings underscore the need for future research to explore gender-specific pathways linking PLEs and distinct forms of violent behavior, as well as the potential moderating role of social and contextual factors.

This study has certain limitations. First, the use of self-reported questionnaires for PLEs and violent behavior data may introduce recall bias, potentially affecting the accuracy of the observed associations. The frequency and intensity of the behavior, as well as the presence of other types of violent behavior, were not assessed, limiting the detailed interpretation of the findings. Future research should incorporate these aspects for more comprehensive data. Second, the longitudinal surveys were conducted in a combined junior and senior high school in Tokyo, Japan, which may limit the generalizability of the findings to other populations. Third, the annual survey intervals may have been too long to capture the rapid developmental changes that occur during adolescence, potentially missing short-term fluctuations in PLEs and violent behavior. Finally, the study did not assess some relevant risk factors for violent behavior, such as impulsivity, mood instability, and traumatic experiences [56, 57], which are also linked to PLEs in prior research [22, 58, 59]. In addition, potential covariates like depression and anxiety symptoms [41, 60]were not included in the RI-CLPM analysis, which may limit the comprehensiveness of the findings and obscure key mechanisms driving the association between PLEs and violent behavior.

In conclusion, PLEs and violent behavior, especially towards objects, may have a bidirectional relationship at both between-person and within-person levels in adolescents. PLEs may increase the risk of later violent behavior, while violent actions could indicate the presence of PLEs, particularly in girls. Screening for PLEs may contribute to early identification of at-risk adolescents, but further research is needed to establish whether such efforts can effectively prevent violent behavior. Similarly, addressing the mental health of adolescents exhibiting violence may help mitigate severe outcomes, although additional evidence is needed to clarify the mechanisms involved.

## Data Availability

No datasets were generated or analysed during the current study.
